# Validation of the ${\text{Pinnacle}}^{3}$ photon convolution‐superposition algorithm applied to fast neutron beams

**DOI:** 10.1120/jacmp.v14i6.4305

**Published:** 2013-11-04

**Authors:** Alan M. Kalet, George A. Sandison, Mark H. Phillips, Upendra Parvathaneni

**Affiliations:** ^1^ Department of Radiation Oncology University of Washington Medical Center Seattle WA USA

**Keywords:** neutron dosimetry, neutron physics, gamma rays

## Abstract

We evaluate a photon convolution‐superposition algorithm used to model a fast neutron therapy beam in a commercial treatment planning system (TPS). The neutron beam modeled was the Clinical Neutron Therapy System (CNTS) fast neutron beam produced by 50 MeV protons on a Be target at our facility, and we implemented the ${\text{Pinnacle}}^{3}$ dose calculation model for computing neutron doses. Measured neutron data were acquired by an IC30 ion chamber flowing 5 cc/min of tissue equivalent gas. Output factors and profile scans for open and wedged fields were measured according to the Pinnacle physics reference guide recommendations for photon beams in a Wellhofer water tank scanning system. Following the construction of a neutron beam model, computed doses were then generated using 100 monitor units (MUs) beams incident on a water‐equivalent phantom for open and wedged square fields, as well as multileaf collimator (MLC)‐shaped irregular fields. We compared Pinnacle dose profiles, central axis doses, and off‐axis doses (in irregular fields) with 1) doses computed using the Prism treatment planning system, and 2) doses measured in a water phantom and having matching geometry to the computation setup. We found that the Pinnacle photon model may be used to model most of the important dosimetric features of the CNTS fast neutron beam. Pinnacle‐calculated dose points among open and wedged square fields exhibit dose differences within 3.9 cGy of both Prism and measured doses along the central axis, and within 5 cGy difference of measurement in the penumbra region. Pinnacle dose point calculations using irregular treatment type fields showed a dose difference up to 9 cGy from measured dose points, although most points of comparison were below 5 cGy. Comparisons of dose points that were chosen from cases planned in both Pinnacle and Prism show an average dose difference less than 0.6%, except in certain fields which incorporate both wedges and heavy blocking of the central axis. All clinical cases planned in both Prism and Pinnacle were found to be comparable in terms of dose‐volume histograms and spatial dose distribution following review by the treating clinicians. Variations were considered minor and within clinically acceptable limits by the treating clinicians. The Pinnacle TPS has sufficient computational modeling ability to adequately produce a viable neutron model for clinical use in treatment planning.

PACS numbers: 87.53 Bn, 28.20.Pr, 87.53.Bn

## I. INTRODUCTION

Interest in the use of high linear energy transfer (LET) fast neutrons for cancer radiotherapy arose from radiobiological experiments in the 1970s that showed advantageous differences in sensitivity, oxygen enhancement ratio, and sublethal damage repair parameters compared to low LET standard X‐ray treatments.^(^ ^1^ ^,^ ^2^ ^)^ To date, the promise of a higher radiobiological effectiveness of neutrons on tumors relative to normal tissues has been clinically proven only for a few tumor types that are radioresistant to low LET radiation, such as salivary gland malignancies^(3)^ and high‐risk soft tissue sarcomas.^(^ ^4^ ^,^ ^5^ ^)^ Clinical trials for advanced prostate cancer illustrated the need for a fully rotational gantry and multileaf collimator in order to avoid higher levels of complications.^(^ ^6^ ^,^ ^7^ ^)^ Otherwise, published clinical trials have shown little benefit from fast neutron radiotherapy. These results, coupled with the substantial cost of such facilities, have left it as a niche treatment, with very few operating facilities worldwide available for clinical use. Consequently, this has restrained commercial development of computerized neutron beam treatment planning systems because there is almost no market incentive.

The fact that neutrons are uncharged and so an indirectly ionizing radiation results in dose deposition characteristics that are similar to photons. Our facility hosts the Clinical Neutron Therapy System (CNTS) cyclotron which generates fast neutron beams having depth‐dose characteristics beyond $D_{\text{max}}$ similar to a 4 megavolt (MV) X‐ray beam. This similarity led to the application of the tissue‐phantom ratio method for dose computations^(^ ^8^ ^,^ ^9^ ^,^ ^10^ ^)^ in the in‐house‐developed University of Washington (UW) treatment planning system (TPS), Prism.^(11)^ This methodology proved to be adequate, since most of the calculations involved interpolations between measured values. Prism was originally designed as a photon and electron beam TPS. However, differences in the magnitudes of scattering and radiation transport meant that the algorithm designed for photons was not as accurate for fast neutrons.

In widely distributed commercial planning systems such as Pinnacle and Eclipse, pencil beam and dose kernel methods^(12)^ are used with convolution algorithms^(^ ^13^ ^,^ ^14^ ^,^ ^15^ ^)^ to compute dose. However, the systems with a model‐based approach to dose computation generally do not possess a computation algorithm specifically designed for fast neutrons (e.g., the Monte Carlo generated dose kernels are specific to photon dose deposition distributions only). Despite this, the fact that neutrons and photons are indirectly ionizing radiations due to being uncharged leads to similarities in their PDD curves and the potential that photon dose algorithms may be used for neutron beam dose computation.

The motivations for this work were to remove the need for maintaining and upgrading the in‐house‐developed Prism TPS and to access a more user‐friendly commercial TPS having interfaces and planning tools with potential for image fusion, intensity‐modulated radiation therapy (IMRT), and X‐ray computed tomography (CT) image‐based dose deposition correction for patient tissue inhomogeneities. The commissioning results presented in this article support the use of neutron beam models of dose deposition derived from modifications to those developed for standard therapeutic energy photon beams.

## II. MATERIALS AND METHODS

Radiation beam dose measurements were made for the fast neutron beam generated by the University of Washington CNTS. This fast neutron beam is produced by a 50 MeV proton beam incident on a beryllium‐copper target. Approximately 25 MeV of energy is lost in the Be target, and the remainder in a copper moderator used to reduce the beam's low energy neutron component.^(^ ^16^ ^,^ ^17^ ^)^ The beam is delivered through a rotating gantry with a source‐to‐axis distance (SAD) of 150 cm. Beam shaping is accomplished by a 40 leaf MLC with a maximum opening of $28.8\times 32.8\;{\text{cm}}^{2}$.

### A. Data acquisition and setup

The measured data for the ${\text{Pinnacle}}^{3}$ TPS version 8.0m (Philips Healthcare, Andover, MA) commissioning were acquired according to AAPM Report No. 7^(18)^ with the Blue Phantom (IBA Dosimetry, Bartlett, TN) scanning system using a fixed step width and an IC30 ion chamber (IBA Dosimetry; Bartlett, TN) flowing 5 cc/min of tissue‐equivalent gas.^(19)^ Most measurements were made in trigger pulse mode for 2000 monitor steps each corresponding to 1.0 second dwell time at 60 MU/min dose rate, and ionization readings were converted to absolute dose via the nominal $10\times 10\;{\text{cm}}^{2}$ calibration field. All point‐dose measurements reported are composed of an average across three electrometer readings, with standard deviations on the order of 0.5%. The IC30 chamber has been shown to be adequate for measuring all neutron beam data (profiles, depth doses, relative output factors, and wedge factors) down to a field size of $2.8\times 2.8\;{\text{cm}}^{2}$.^(20)^

Output factors and profile scans for open and wedged fields were measured according to the Pinnacle physics reference guide recommendations for photon beams. Source‐to‐surface distance (SSD) was set at 150 cm for both output factors and profiles, and depth doses were measured from the water phantom surface to a 30 cm depth in at interval steps of 0.5 cm. Scan length used for field sizes from 2.8 to 10.3 cm^2^ was 0.2 step size and 0.50 for fields 12.8 cm^2^ or larger. Irregular field profiles were also measured in trigger pulse mode with scanning parameters equivalent to those with similar field sizes.

To evaluate the robustness of the collected commissioning data, PDDs and calibration outputs measured several years apart were compared as various field data were tested and acquired. The PDD has changed little for this treatment device over a decade, with the maximum difference between measurements over that period being 1.7%.

### B. Machine characterization

**Figure 1:**
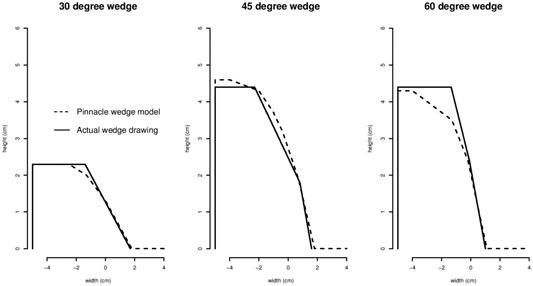
Overlay of physical wedge profiles used in Pinnacle versus the actual cyclotron wedge profile drawings.

The cyclotron machine was created in the Pinnacle TPS machine database and edited as a type “Other” machine with fixed jaws and MLC‐only beam shaping to match the actual physical characteristics of the machine a closely as possible. The cyclotron, in fact, does not have any jaws. Most other physical machine parameters (e.g., gantry rotation angles, leaf widths, wedges) were entered similarly to other currently existing medical linear accelerators at our facility. Physical wedges were initially created according to the actual design geometry derived from the machine manufacturing blueprints. In the course of the neutron beam modeling process, both the geometry and assigned physical material density coefficients had to be modified to create a closer agreement between the measured wedge data and the calculated Pinnacle model (Fig. 1). Some of the differences between the actual physical wedge and the model wedge may be explained by the fact that the cyclotron beam direction from target to wedge is incident on the flat base of the wedge, while in Pinnacle the beam incidence is restricted to being upon the sloped surface of the wedge. However, it is suspected that this effect might have less to do with wedge orientation and more to do with the difference in the neutron kerma factors for the neutron spectrum being quite different from the mass attenuation coefficients of the photon spectrum used in the Pinnacle physics model.

#### B.1 Small fields vs. large fields

The cyclotron gantry head also contains a unique flattening filter set. It has a built‐in selection system that is based on field size. This was installed to match beam flatness specifications. The small field size (SFS) filter is automatically selected by the machine control software for field sizes smaller than or equal to $12.8\times 12.8\;{\text{cm}}^{2}$. The large field size (LFS) filter is automatically selected by the control system for field sizes larger than $12.8\times 12.8\;{\text{cm}}^{2}$. What size filter chosen is defined not by square (or equivalent square) field size but by the maximum leaf separation beyond the central axis of the field (i.e., greater than 6.4 cm leaf separation in any of the MLC principal axis directions from central axis). Beam spectra and profiles were different enough between the small and large filters that the creation of separate machine models in the Pinnacle system was required for each size of filter to satisfy our model accuracy acceptance criteria (Table 1). To complete a dose calculation in Pinnacle, an output factor is chosen based on the equivalent square of the unblocked MLC field. Therefore, additional output factors were required for small field sizes with the large filter in place to account for MLC shapes that meet both the CNTS criterion for large fields and the Pinnacle criterion for small fields. The Pinnacle system has no practical way to select the correct beam model based on the selection rules that govern the machine. We constructed, therefore, two separate machines in Pinnacle — a “large field” machine and a “small field” machine.

**Table 1:** Acceptability criteria for external beam dose calculations

*Situation*	*Central Axis (%)*	*Inner Beam (%)*	*Penumbra (%)*	*Outer Region (%)*
Square fields	2	2	5	5
Rectangular fields	2	2	5	5
Wedged fields	2	2	5	5
MLC‐shaped fields^a^	−	−	−	−
Slab inhomogeneities^a^	−	−	−	−

a Accuracy equivalent to or better than current treatment planning system, Prism.

### C. Pinnacle modeling and parameter optimization

Dose distribution data were input to the Pinnacle system via the Wellhofer automated dosimetry system database (IBA Dosimetry America, Bartlett, TN, U.S.A.). Relative output factors for both the large field and small field machine were generated by normalizing to the $10.3\times 10.3\;{\text{cm}}^{2}$ open field at 10 cm depth using the small field filter. The calibration geometry used was a SAD setup for 10 cm depth in water with 1 cGy/MU is set to 1.7 cm ($D_{\text{max}}$) at 150 SAD.

The Pinnacle modeling algorithm offers two main modeling methods and a series of optimization steps. First, one can choose either an “all field sizes” or “each field size independently” model. The “all field sizes” model attempts to choose one physics model optimized to all field sizes, and the “each field size” model must be performed for each measured field size in the dataset. It is much more effort to model each field size, so an extensive test of the “all field sizes” modeling process was performed initially. The results of this attempt did not meet the acceptability criteria outlined in Table 1 for all open square fields, so was rejected. The neutron beam dose is significantly different to photon dose in both the buildup region and in the deep scatter off‐axis region. The one‐size‐fits‐all model could not meet the commissioning acceptance criteria of percentage errors less than 2% in high‐dose regions (inner beam). We were forced, then, to use an independent model for each open field size and all measured field sizes for each wedge. This successful procedure for modeling is described in the following section.

#### C.1 Spectrum building and depth dose

For each field size, the FineTuneSpectrum optimization routine was run first to match the depth dose. The electron contamination was turned off, as this only served to add dose where the profile was already too high. In Pinnacle, the user is required to set a fixed depth level (point of connection) where the measured and modeled data must have the same dose. The point of connection was set differently for many of the field sizes, as needed, and was often placed in the buildup region to get a closer match overall. A general criterion for acceptance was less than 2% error for points in the depth range of 1.0 to 30.0 cm. It was not possible to match the model to the data within this dose error criterion for the first 0.5 cm depth from the surface due to the restriction imposed by the Pinnacle beam model parameters.

#### C.2 Off‐axis model and beam fluence

The FineTuneCrossBeam optimizer was then run with an arbitrary profile to fit the field shoulders and penumbra region at the edge of the field. A manual adjustment of the arbitrary profile was made to fit the profile off‐axis regions. Iteration of individual automodeling steps from this sequence and manual adjustments were performed to obtain 2% or better agreement between the model and data in the high‐dose region and shoulders of the profile, and 5% in the penumbra and low‐dose profile tails.

#### C.3 Wedge modeling

The initial model obtained by the all‐wedge field modeling procedure did not produce a 2% or better dose agreement between the model and data in the high‐dose region along the wedge angle, and 5% in the penumbra and low‐dose profile tails beyond the heel and toe of the wedge. Iterations of the beam model optimization routine and adjustments to the wedge physical model were run until the predicted shape of the dose distribution gradient in water was fairly close to the measured data. Profiles were then computed with the FineTuneAllForWedge optimization sequence. Dose match was still not meeting the threshold criteria at this level, so FineTuneCrossBeam was run to get the nonwedged direction of the field to match. A fine‐tuning of the arbitrary profile was then performed until the dose match of 2% or better was achieved and could no longer be improved.

**Figure 2:**
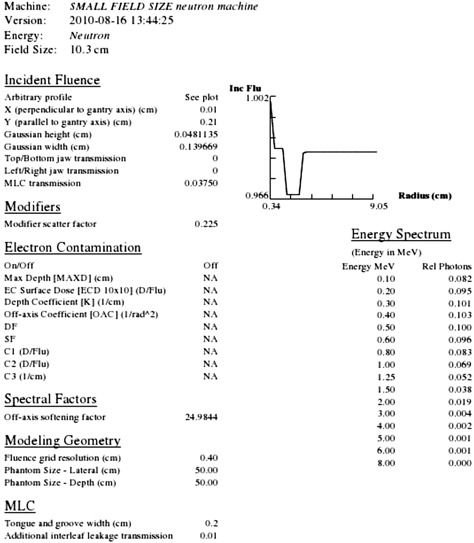
Example Pinnacle beam modeling parameters for the 10.3×10.3 field.

Beam spectra were fairly similar within each machine type even though a unique model for each field size and wedge combination was used. Spectra generally peaked between 0.5–0.8 MeV with a FWHM (asymmetric) spread of ~0.4MeV. The resulting model parameters for the $10.3\times 10.3\;{\text{cm}}^{2}$ (calibration) field are shown in Fig. 2.

### D. Dose calculations

**Figure 3:**
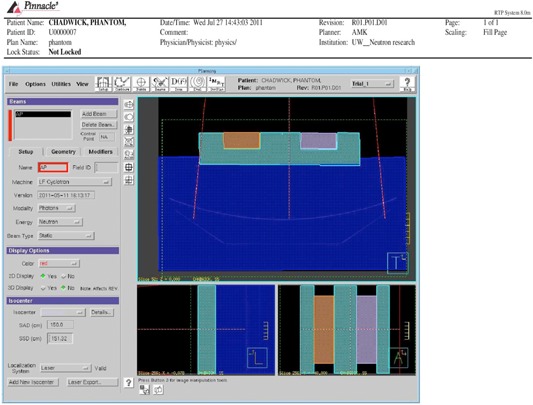
A heterogeneous density phantom as seen in the Pinnacle planning system. Rectangular acrylic rods were inserted into Styrofoam block cutouts to create stepwise high‐gradient density changes in the beam path.

Doses were computed for comparison by creating 100 MU beams in Pinnacle incident on a water‐equivalent phantom for open and wedged square fields, as well as MLC‐shaped irregular fields in a 150 SSD setup at 0.2 mm grid spacing and $40\times 40\times 40\;{\text{cm}}^{2}$ grid volume. Pinnacle generated dose profiles and central axis dose points were compared to doses computed with the Prism treatment planning system in the same geometry as the Pinnacle setup and also planned for a 100 MU beam. Point dose depths between 1.7 cm and 20.0 cm are compared and wedge profiles depths of 5 cm, 10 cm, and 15 cm are compared. A separate phantom was constructed from Styrofoam blocks and acrylic rods (Fig. 3) to examine dose calculation accuracy in this simple heterogeneous phantom. The device was positioned above the water phantom, and profile measurements were taken below it at depths of 5, 10, and 15 cm for an open $28.8\times 32.8\;{\text{cm}}^{2}$ field. Given that the vast majority of clinical tumor treatments are in head and neck anatomic locations such as the nasopharynx or parotid where total anatomic diameter rarely exceeds 20 cm, we find these depths to be the most clinically relevant to examine. We also compared to point doses measured in a water tank. Percentage dose differences are calculated using the following standard formula

(1) $$100\%\times (D_{P}-D_{C})/D_{C}$$

where $D_{P}$ is the Pinnacle computed dose and $D_{C}$ is the comparison dose, either Prism‐computed dose or measured value. To make the dosimetric test more stringent, we did not normalize the dose at any point for any of the beams.

### E. Clinical Comparisons

For several clinical cases, a matching Pinnacle plan was created with the same wedges, MUs, dose points, and site setup for comparison in more complicated situations with inhomogeneous media and overlapping irregular fields. Doses were computed in each TPS and the dose at various points of interest, target coverage, and normal structure doses were examined and compared by experienced clinicians treating these patients. These clinical comparisons are of particular importance because of their relevance to the known clinical outcomes based on our extensive practical experience with the Prism TPS. Since the fast neutron treatment beam at our facility is unique even compared to the two other neutron treatment facilities in the USA, correlation of clinical outcomes using Prism is essentially the only landmark by which to judge the prescriptions currently used and the ways in which they might change by using a system that shows different isodose lines, monitor units, and predictions.

We compared computed doses between the Prism and Pinnacle treatment planning systems. Clinical cases are considered exclusively here. We present two separate comparisons: 1) a dose point comparison between Prism and Pinnacle doses both computed on the same full CT‐based structure sets derived from a historic clinical patient database, and 2) dose point comparisons of clinically planned beams computed in Pinnacle compared to Prism‐computed doses and measured doses in phantom conditions.

Eight cases were selected from our clinical database (Jan 2011‐June 2011). Most cases were head and neck tumor treatments, and planning target volumes ranged from 13 to 1033 cm^2^. Some treatments involve supraclavicular and half‐beam block fields, as well as junction changes and boost fractions. Most plans also involved the use of multiple wedges and both large and small fields, as previously defined. Dose points were not created specifically for comparison; rather, the dose points examined here were created by dosimetrists during the planning process for use in evaluating areas of concern either in the GTV, PTV, or critical surrounding structures. This was in keeping with our historic clinical practice and plan evaluation process using Prism. For each Prism plan, the corresponding beams, prescriptions, and dose points were recreated in the Pinnacle TPS. It was our common practice of convenience to draw targets and organs at risk in Pinnacle and import those structures to Prism for dose calculation; so, there was no difficulty in obtaining identical geometries in both systems. Prescriptions were designated by giving the same MU and fractionation scheme to each beam exactly as found in Prism. Dose was computed using the Pinnacle physics model for neutrons and point doses were examined and analyzed. The use of distance to agreement^(21)^ is also considered when evaluating pointdose errors. Prism dose was used as a measure of our traditionally acceptable quality for most regions of interest and, therefore, as a basis to validate the Pinnacle model in a clinical context through this intercomparison.

### F. Acceptability criteria

All efforts were made to assure that the modeling of the neutron data in the Pinnacle treatment planning system satisfied TG53‐based criteria^(22)^ for the acceptance of a treatment planning system and the modeling QA that followed was extensive. Clinical acceptance criteria were evaluated separately from the TPS modeling criteria identified in the previous section. We considered the essential criteria for clinical acceptability as accuracy which met or exceeded that of Prism since this TPS has been in clinical use for neutron beam therapy for more than a decade. Historically, we used an acceptance threshold for agreement between the Prism point dose and MU second‐check dose at differences of 5% but because the difference between the Pinnacle and Prism dose algorithms is significant, we also incorporate some level of clinical judgment with respect to differences which exist between the planning systems. In general, the error tolerance we use for neutron therapy is somewhat larger than our tolerance for photon therapy. Knowledge regarding radiobiological effect and differences in scattering of neutrons in various tissue types is not as comprehensive as it is for photons and, therefore, cannot be accounted for well in treatment planning systems. The outcomes of neutron treatment then rely more heavily on previous experience delivering monitor units calculated in the Prism system and its software precursors.

## III. RESULTS

In this section, we compare Pinnacle‐computed dose predictions to measurement and Prism computation. Most data are presented in tabular form as doses along the central axis. Much of the current clinical treatment involves the use of multiple wedges so those fields are presented graphically, as they are more easily examined by inspecting profiles across the wedged portion of the field. For irregular fields and clinically used fields, the beam's eye view (BEV) is also given to provide clarity. An extensive list of fields was tested, but we present only a few representative samples in this article.

### A. Small fields

Since the beam model was split into two separate machines, we evaluate the results for small fields and large fields independently, and in this section we consider small fields, followed by the large fields in section B.

#### A.1 Open and wedged fields

**Figure 4:**
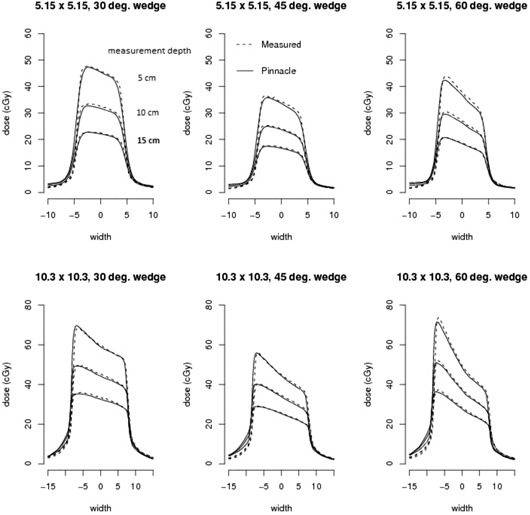
Comparison of dose profiles between measured and modeled neutron data for small square wedged fields at 5.0, 10.0, and 15.0 cm depths.

Pinnacle dose point computations (Table 2) along the central axis of small open fields exhibit discrepancies less than 3.3 cGy (3.2%) from either Prism or measured doses for all depths (1.7 to 20 cm). Wedged field dose differences do not exceed 2.4 cGy (3.8%), 0.8 cGy (1.7%), and 2.0 cGy (3.8%) for the 30°, 45°, and 60° wedges, respectively. We found upon qualitative inspection of the wedged field profile results that the computed dose and the measured dose agree quite well both in the penumbra and the sloped portions of the wedge for depths of 5, 10, and 15 cm (shown in Fig. 4).

**Table 2:** Open square small field doses and dose differences between Pinnacle, Prism, and measured data

*Field Size* $\left({\text{cm}}^{2}\right)$	*Depth(cm)*	*Pinnacle Dose (cGy)*	*Prism Dose (cGy)*	*Measured Dose (cGy)*	*Pinnacle‐Measured (cGy)*	*Pinnacle‐Prism (cGy)*
	1.7	81.3	81.9	81.5	−0.2	−0.6
	3.0	77.7	76.9	77.7	−0.0	0.8
	5.0	68.2	67.7	68.0	−0.2	0.5
5.3	10.0	47.2	47.2	48.4	−1.2	0.0
	15.0	32.5	32.4	33.2	−0.7	0.1
	20.0	22.7	22.5	22.6	−0.1	0.2
	1.7	95.7	96.9	97.6	−1.9	−1.2
	3.0	92.7	92.4	93.4	−0.7	0.3
	5.0	84.2	83.7	84.8	−0.6	0.5
10.3	10.0	63.1	63.0	63.5	−0.4	0.1
	15.0	46.5	46.4	46.6	−0.1	0.1
	20.0	34.1	34.1	33.7	0.4	0.0
	1.7	101.3	104.6	103.8	−2.5	−3.3
	3.0	100.5	100.2	99.5	1.0	0.3
	5.0	91.9	91.7	90.6	1.3	0.2
12.8	10.0	70.0	70.2	70.2	−0.2	−0.2
	15.0	52.7	52.7	52.7	0.0	0.0
	20.0	39.1	39.3	39.1	0.0	−0.2

#### A.2 Irregular fields

**Figure 5:**
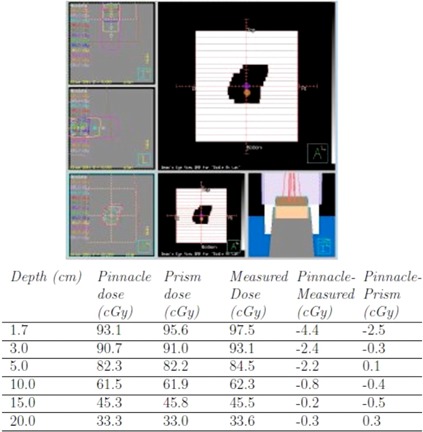
Right lateral femur treatment field and central axis dose comparison with both Prism‐computed dose and measured dose delivered at 150 SSD and 100 MU.

Doses in the water phantom for several small irregularly shaped MLC configurations including “x” shapes, rectangles, and “pear” shapes were computed in Pinnacle and compared to both Prism‐computed doses and measured doses for 100 MU beam delivery. One representative example, shown in Fig. 5, includes the BEV and tabulated dose difference from measured data. Pinnacle predictions can underestimate measured dose at shallow depths by up to 5.1 cGy, for example, in narrow diagonal fields (not shown), and differences of up to 8.6 cGy can occur for shapes which differ significantly from the symmetric square field configuration about central axis.

### B. Large fields

The large field machine case was evaluated with the addition of specialized midline block fields which represent baseline dose expectation for clinically common supraclavicular treatments.

#### B.1 Open and wedged fields

**Figure 6:**
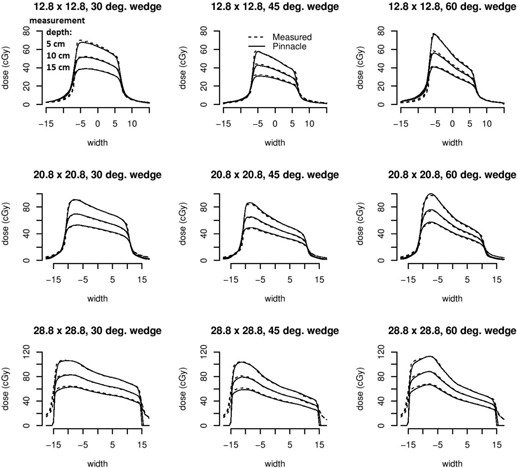
Comparison of dose profiles between measured and modeled neutron data for large square wedged fields at 5.0, 10.0, and 15.0 cm depths.

Pinnacle dose point computations (Table 3) along the central axis of large open fields exhibit discrepancies of less than 3.3 cGy (3.3%) from either Prism or measured doses for all depths (1.7 to 20 cm). Wedged field dose differences do not exceed 3.9 cGy (4.3%), 2.5 cGy (4.4%), and 2.3 cGy (3.2%) for the 30°, 45°, and 60° wedges, respectively. Wedged large field profile results of the computed dose also agree quite well qualitatively both in the penumbra and the sloped portions of the wedge for depths of 5, 10, and 15 cm (shown in Fig. 6).

**Table 3:** Open square large field doses and dose differences between Pinnacle, Prism, and measured data

*Field Size* $\left({\text{cm}}^{2}\right)$	*Depth (cm)*	*Pinnacle Dose (cGy)*	*Prism Dose (cGy)*	*Measured Dose (cGy)*	*Pinnacle‐Measured (cGy)*	*Pinnacle‐Prism (cGy)*
	1.7	110.4	111.4	109.6	−0.8	−1.0
	3.0	106.4	107.0	107.7	−1.3	−0.6
	5.0	97.9	98.5	99.3	−1.4	−0.6
16.8	10.0	76.3	77.1	77.2	−0.9	−0.8
	15.0	58.8	59.0	59.5	−0.7	−0.2
	20.0	44.4	45.0	45.0	−0.6	−0.6
	1.7	116.0	117.7	117.0	−1.7	−1.7
	3.0	111.8	113.1	112.9	−1.1	−1.3
	5.0	103.7	104.9	104.3	−0.6	−1.2
20.8	10.0	82.7	83.1	82.7	0.0	−0.4
	15.0	64.8	64.6	64.1	0.7	0.7
	20.0	49.7	50.1	49.2	0.5	−0.4
	1.7	124.4	127.5	127.0	−2.6	−3.1
	3.0	121.6	123.1	122.7	−1.1	−1.5
	5.0	113.4	114.3	113.7	−0.3	−0.9
28.8	10.0	91.4	92.1	91.5	−0.1	−0.7
	15.0	72.8	72.4	72.5	0.3	0.4
	20.0	57.2	56.6	56.6	0.6	0.6

#### B.2 Irregular fields

In the large field model, we were able to explore Pinnacle dose comparisons for more elaborately shaped irregular fields such as “L” shapes and off‐axis squares, as well as “C"‐shaped configurations and midline blocks (7, 8). In the “C” configuration the central axis is completely blocked, but differences between Pinnacle and measured dose in the open portion of the field exhibit discrepancies of less than 3.5 cGy (3.0%). Dose difference between Pinnacle‐computed dose and measured dose in the open portion of the off‐axis square and “L"‐shaped fields was not greater than 0.3 cGy (0.3%) and 2.7 cGy (2.6%), respectively.

Cross‐plane dose profiles across the midline block in the supraclavicular field configuration measured at 5, 10, and 15 cm depths compared with Pinnacle‐computed dose are shown in Fig. 9. Dose agreement in the outer penumbra and under the midline block was found to be better than in the open portions of the field, where the shape of the dose distribution is less accurate. Comparisons of the Pinnacle profile to the Prism profile show that Pinnacle accuracy is on par with Prism's in these cases.

**Figure 7:**
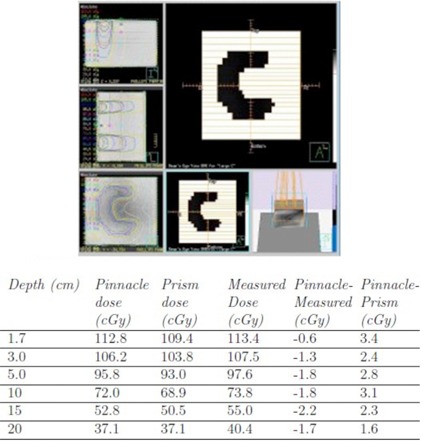
Large C‐shaped field comparison between Pinnacle, Prism, and measured dose delivered at 150 SSD and 100 MU. Points examined are off‐axis laterally by 7 cm towards the open portion of the field.

**Figure 8:**
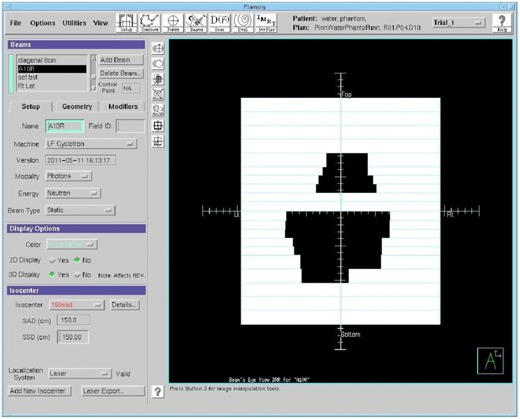
Beam's eye view (BEV) of midline block test field for dose profile comparisons.

**Figure 9:**
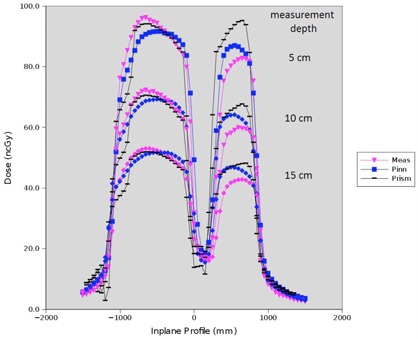
Dose profiles under the midline block. Measured and Pinnacle‐computed doses shown for 5.0, 10.0, and 15.0 cm depths.

### C. Clinical Results

The point label and 3D location of each dose point was recorded in a spreadsheet, along with the Pinnacle dose and Prism dose. (A subset is shown in Table 4.) The absolute dose difference and percentage dose difference are computed on the far two right‐hand columns. A total of eight cases representing 107 points was examined in this intermediate analysis. Several points (8) were identified as outliers (> 4 standard deviations from mean) and were removed from this analysis. Each outlying point removed had a low relative dose to the given structure, which caused a large percentage error overall.

**Figure 10:**
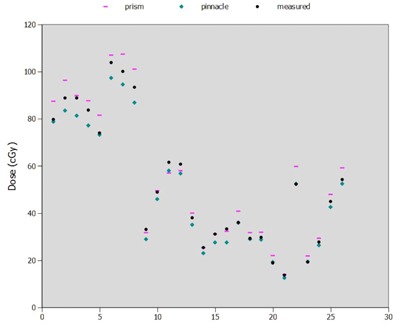
Dose points from Pinnacle, Prism, and measurement based on clinical fields which exhibit greater than 7% discrepancy in clinical MU second checks. Measurements here were taken in a water‐equivalent plastic stack specially designed to mimic neutron TPR measurements in water. Abscissa units are arbitrary and used here only for indexing.

For the 12‐month period during which each clinical neutron radiation therapy patient was planned in both treatment systems, we also instituted a measurement protocol for the beams which exhibited large discrepancy (error>7%) between Pinnacle's and Prism's standard MU second‐check calculation tool. The MU second check computes the MU difference when beams are incident perpendicularly to the surface of a uniform slab phantom. For 13 patients, we measured 26 fields whenever the predicted MU discrepancy between Pinnacle and Prism exceeded 7%. These fields comprised off‐axis, wedged, and heavily blocked fields (both LF and SF). We delivered these fields to a uniform slab water‐equivalent plastic stack phantom with the Pinnacle‐computed MU. Expected doses ranged from 12.5 to 97.3 cGy. Absolute doses from Pinnacle, Prism, and measurement are shown in Fig. 10. The mean dose difference and standard deviation between Pinnacle and measurement in these cases was −5.6±4.4cGy, compared to the Prism difference from measurement of 5.2±6.2cGy. That is, in most cases, Prism overestimated the dose compared to measurement, while Pinnacle underestimated the dose, in most cases. Large discrepancy between Pinnacle and Prism, therefore, represents mainly a difference in the way each treatment planning system handles irregularly shaped off‐axis wedged fields more than it does large actual disagreement from real measured dose.

**Figure 11:**
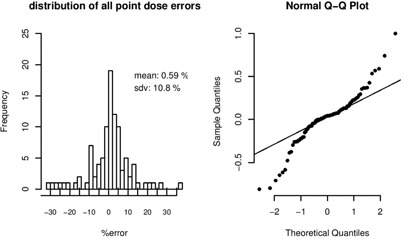
Dose point error histogram (left) and quantile comparison with Gaussian “normal” distribution (right). Dose point errors are random to within 1SD.

We examined the remaining Pinnacle‐computed dose points in the CT structure set conditions in terms of the percentage dose difference from Prism. Both the magnitude and the frequency of error are considered in determining quality of the Pinnacle dose computation with respect to Prism. The distribution of errors in the dose point comparison gives us a measure of whether the errors are derived from random processes or if there is some systemic difference between the two TPS dose computations. The average error of all dose points is 0.59%, which is quite a reasonable agreement given the different nature of computation algorithms between the two planning systems and the tendency of each system to produce systematic errors in opposing dose direction, as seen previously in the measurement analysis (Fig. 10). However, the width of the distribution is somewhat less impressive. In Fig. 11, although the errors appear “normally” distributed, the quantile comparison on the right suggests that the tails of the distribution are too great in magnitude to imply pure random noise processes. Even with the outliers removed, there are many dose points (23 out of 97) with poor agreement (error>10%). While a 10% dose difference may be debated as a threshold for poor agreement, it is important to impose another threshold for quality, since any given dose point examined in a clinical plan may be used for medical decision‐making. In fact, there are points in this analysis at which Prism dose is below the recommended normal tissue complication probability^(23)^ of 5% within five years $\left({\text{TD}}_{5/5}\right)$ dose constraint limit for a particular structure (i.e., temporal tip), while the Pinnacle dose is slightly above, but within clinical acceptance. Some points lie in high gradient areas where significant dose difference is not unexpected over a short distance range. For example, the point in Table 4 (Inf 1265 PTV) lies on the border between the 1265 and 1840 PTVs. Boost fields are given just adjacent (superior) to this point to bring the dose up to the 1840 PTV, creating a dose gradient around 63 cGy/mm in this region. Distance to agreement for this point is 3.5 mm, even if the disagreement is closer to 18% at the location.

**Table 4:** Clinical dose point location and absolute dose comparison

*Point Name*	*Pinnacle Dose (cGy)*	*Prism Dose (cGy)*	*Dose Difference (cGy)*	*Dose Differ (%)*
Iso	1795.3	1840.0	−44.7	2.4
c1 cord	551.6	551.0	0.6	−0.1
c2 cord	583.3	592.1	−8.8	1.5
c3 cord	661.5	661.0	0.5	−0.1
c4 cord	702.3	697.0	5.3	−0.8
sup 1265 PTV	1343.2	1306.0	37.2	−2.8
sup 1840 PTV	1942.5	1915.0	27.5	−1.4
ant 1840 PTV	1894.7	1929.0	−34.3	1.8
post 1840 PTV	1931.7	1939.0	−7.3	0.4
ant 1840 PTV2	1935.6	1968.0	−32.4	1.6
post 1840 PTV2	1916.8	1926.0	−9.2	0.5
Inf 1840 PTV	1907.9	1874.0	33.9	−1.8
Inf 1265 PTV	1815.3	1540.0	275.3	−17.9

### D. Output Factor Selection

This section examines the apparent problem of poor output factor selection by the Pinnacle TPS in thinner fields which are diagonal with respect to the leaf motion direction. The Pinnacle photon model attempts to account for the blocking by computing a phantom scatter, and then correcting for the headscatter using measured data. The Relative Output Factor for an open field is calculated using the equation:

(2) $$D_{\text{FS}}/D_{\text{CFS}}$$

where $D_{\text{FS}}$ is the measured dose for the given field size and $D_{\text{CFS}}$ is the measured dose for the calibration field.^(24)^ Prism suffers from a similar problem which stems from the use of a 4Area/Perimeter (4A/P) estimation of the equivalent square field used for output factor lookup, independent of the Clarkson scatter integration type methods used for dose computation in nonrectangular fields. Pinnacle also uses the 4A/P method for the overall dose scaling, but it is not obvious if the effect is comparable to Prism. We include here a short description of the effects of field rotation in Pinnacle and aspect ratio in an attempt to better quantify this issue and account for it in clinical applications.

#### D.1 Rotation effect

**Figure 12:**
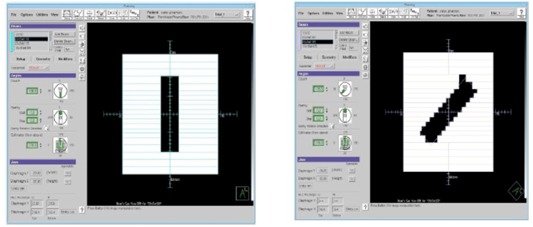
BEV for two rotated fields having the same equivalent square size.

A $20.8\times 5\;{\text{cm}}^{2}$ rectangular field was constructed in Pinnacle and computed on an arbitrary water phantom. The MLC shape of the beam was tied to a block on a $20\times 5\;{\text{cm}}^{2}$ contour to ensure that the outlined structure remained fixed, and the Pinnacle system was allowed to autocontour this structure with a MLC‐based block for varying degrees of couch rotation (Fig. 12). Therefore, the field size, equivalent square field area, and output factor should remain almost constant with collimator rotation.

**Figure 13:**
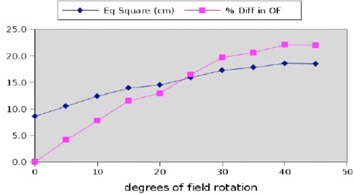
Output factor and equivalent square dependency on rotation. Small changes in the orientation of a skinny field can cause large changes in the Pinnacle‐computed output factor. The ordinate axis here is dual unit (degrees rotation and percent difference).

The effect on Pinnacle‐calculated equivalent squares and output factors used for dose computation is examined below for such rotations, and the results are an increase in the calculated equivalent square and an associated increased output factor (Fig. 13). This large difference translates directly to dose. Measured data indicate that the increase in computed dose is incorrect and that actual doses for rotated rectangular fields are much lower than computed by Pinnacle. The effect is very large in neutrons for which the output factor for a $30\times 40\;{\text{cm}}^{2}$ field is 46% larger than the calibration field $\left(10\times 10\;{\text{cm}}^{2}\right)$. By contrast, the $40\times 40\;{\text{cm}}^{2}$ field output factor for a 10 MV photon beam generated by an Elekta Precise linac (Elekta AB, Stockholm, Sweden) is 12% larger than the calibration field $\left(10\times 10\;{\text{cm}}^{2}\right)$. It should be noted that although this is an error for photon beams as well as neutrons, this error is corrected for in the final computation for photons but not neutrons.

#### D.2 Field length effect

**Figure 14:**
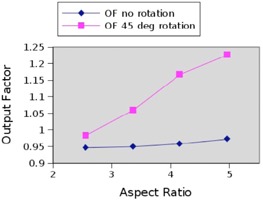
Output factor dependency on rectangular field length in natural (along or orthogonal to MLC motion direction) and diagonal orientations.

The Pinnacle system chooses an output factor based on the area of the smallest square that circumscribes the open field and is also aligned perpendicular with the MLC leaf directions.^(25)^ This effect should naturally be larger for a long skinny field than a short one. To show how this affects neutron dose computation, we also computed the output factor for a standard field versus a 45° rotated field of 5 cm width but variable length. Given the results above, this example constitutes a worst‐case rotation scenario. The results verify that the computation error for long thin fields is greater with rotation. Figure 14 shows the predicted output factor change with respect to aspect ratio (field length) for a rotated and nonrotated field. As the rotated field gets longer, the output factor increases significantly beyond that of a nonrotated field. For rectangular fields beyond $12.8\times 5\;{\text{cm}}^{2}$, the difference in output factor for the rotated field is above 5%.

### E. Inhomogeneous media

**Figure 15:**
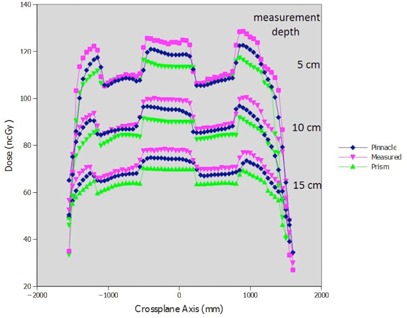
Dose comparison of Pinnacle, Prism, and measurement in heterogeneous phantom conditions at depths of 5, 10, and 15 cm in water.

Pinnacle dose profiles computed at depths in water underneath the heterogeneous phantom are compared to measurement and Prism doses (Fig. 15). In this case, both Pinnacle and Prism underestimate dose compared to measurement; however, Pinnacle accuracy compared to measurement is better than that of Prism for all depths tested in the unblocked portion of the field.

## IV. DISCUSSION

Comparison of Pinnacle dose predictions to Prism's and to measured doses meet or exceed the criteria outlined previously for the extensive set of conditions examined in this article. Comparisons to the measured doses in both the standard commissioning conditions, irregular field shapes, and clinical case comparisons indicate that the Pinnacle dose model reproduces isodose distributions of sufficient accuracy for 3D conformal patient planning. In the most extreme cases such as the large field midline block and many of the off‐axis heavily blocked wedged fields, we observe differences in the predicted dose profiles compared to measurement, which may indicate that better precision could be achieved by substituting neutron dose kernels for the photon dose kernels in the model. However, the aggregate dose difference is still within our historical clinical acceptance of accuracy for such fields generated by Prism for neutron beams. In some situations, Pinnacle provided dose distribution information that was lacking with Prism. For example, at field junctions Prism underestimated dose in the transition region. However, in our clinical experience that included “feathering the junction” after every 5–7 fractions, there were no recurrences or match‐line “hot spot” fibrosis at these junctions. The dose distributions seen on Pinnacle‐generated plans were more in keeping with our historical clinical expectations. The dose difference which occurs in opposing directions seen in the irregular‐shaped clinical field set can be leveraged to increase the accuracy of treatment planning for specific cases. By using a truly independent second check (Prism point calculation tool) on a Pinnacle generated plan, one can identify fields which are problematic in both systems, thereby allowing changes to be made to the treatment plan prior to delivery (e.g., moving a boost isocenter to avoid excessive blocking near the central axis).

The evaluation of output factor selection also shows Pinnacle's limitations reconciling neutron scatter contribution in the photon scatter model. Pinnacle uses a correction factor to scale the computed fluence into dose. Because the physics model assumes a photon fluence, large corrections are required by the system to scale the output to match the neutron beam. The resulting effect is a systematic overscaling of dose under these conditions, which is not seen in standard photon beam models. Output corrections on the order of 2%‐5% are typically required for clinical beams generated in Prism due to its similar use of the 4A/P method for output factor computation, and those corrections may also be implemented in Pinnacle. However, because Pinnacle is a commercial product with propriety software, we do not have access to its source code to modify the equivalent field size output estimation for MLC blocked fields, such as was done in Prism. We overcame this problem by implementing a wide $\left(50\times 50\;{\text{cm}}^{2}\right)$ zero‐thickness compensator in Pinnacle, which does not modulate the intensity profile but does allow for an output scaling factor to be applied. The implemented correction factors are based on the ratio of output factor (OF) for the original field to the OF for a field with a more appropriate equivalent square computed using the Pinnacle scripting capability.

Another important point to note is the simple approach used to account for neutron attenuation through heterogeneous tissue densities presented by the patient X‐ray CT image information. Currently, density correction is made only for air $\left(0.1\;g/{\text{cm}}^{3}\right)$ and lung tissue $\left(0.3\;g/{\text{cm}}^{3}\right)$ in Pinnacle, as it was historically in Prism. Pinnacle's accuracy was shown to be improved over the Prism TPS in the simple phantom study here, but unlike photons and electrons, fast neutrons scatter primarily from nuclei and not orbital electrons. Therefore dose distribution corrections based on physical density or electron density may not provide accurate attenuation correction for bone and fat tissue. A model presently does not exist which accounts for neutron scatter cross‐sections in specific tissues. Developing such a model for neutron beam planning with Pinnacle will require the tissue‐specific measurements and/or Monte Carlo simulations. More accurate modeling of neutron scattering under heavy blocking is also a potential area of improvement, as is modeling spectral changes to the neutron beams following their attenuation in wedges.

## V. CONCLUSIONS

The photon convolution dose computation algorithm in the commercial treatment planning system Pinnacle may be successfully applied to the neutron beam data. Based on standard commissioning criteria outlined in TG‐53 and also comparison with results generated by an in‐house‐developed treatment planning system named Prism, the Pinnacle photon beam model applied to fast neutrons provides a description of the neutron beam dose distribution data in patients of sufficient accuracy to be suitable for clinical treatment planning.

## ACKNOWLEDGMENTS

The authors would like to acknowledge Ruedi Risler for his invaluable expertise and assistance in dosimetric measurements, and also Ira Kalet and Jon Jacky for their consultation on dose‐calculation algorithms for radiotherapy computing software devices.

## Supporting information

Supplementary Material
