# Supplementary figures and images for: Validation of the Pinnacle3 photon convolution‐superposition algorithm applied to fast neutron beams
Source: J Appl Clin Med Phys. 2013 Nov 4;14(6):133–54. doi: 10.1120/jacmp.v14i6.4305 (PMC5714634; doi:10.1120/jacmp.v14i6.4305)

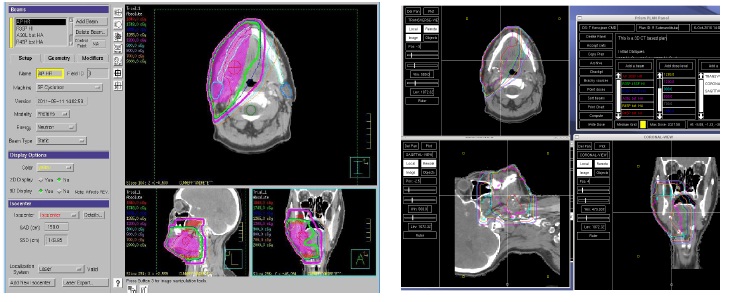

Supplement: Supplementary file 1 — Supplementary Material [file ACM2-14-133-s001.jpg]
